# Editorial

**Published:** 1849-08

**Authors:** 


### THE MEDICAL EXAMINER.

### PHILADELPHIA, AUGUST, 1849.

### CHOLERA AMONG MEDICAL MEN.

Numerous reports of death by cholera among medical men, both in this country and abroad, are reaching us daily. In St. Louis, we understand by the daily prints that several of the "Professors of Medicine" are among the victims; whether these are our friends in the medical schools of that city, we are not credibly informed. In this city we have to record the death of two who have fallen victims to this scourge: Mr. Thomas M. Flint, a valued contributor to this journal, and Mr. S. Warren White, late of Yazoo city, Miss., Students of Medicine. Both of these gentlemen had volunteered their services, among a number of others, during the prevalence of the cholera in the Philadelphia Almshouse, Blockley, the medical officers of which institution have been worn out by their constant devotion to the sick. Both fell victims to the dread disease, in consequence of the fatigues attendant upon their duties. One had but recently graduated, and gave promise of a life of usefulness and distinction; the other was looking forward in bright anticipation of the highest honors of his Alma Mater, after having served with much distinction throughout the Mexican war.

We have often deplored the little impression that is made by the decease of those whose lives are devoted to the cause of humanity. When a medical officer distinguishes himself on the field of battle by cool courage, or falls while in the discharge of his duties, no glowing despatch or public demonstration chronicles his services to an unsympathizing public; in the time of pestilence, when all others are fleeing before its breath, no eulogy of grateful praise is offered to those whose lives are risked, aye often sacrificed, to stay its progress; and those who fall, as these have fallen, too often sink into the grave, followed only by the tears and regrets of the few who knew their worth.

—

By our foreign exchanges we learn the death of Prof. Blandin, in the fiftieth year of his age; of M. Bourgery, author of L'Anatomie de l' Homme; M. Boudet, member of the Academy of Medicine, and M. Mojon, of Geneva, an eminent surgeon under Napoleon, and long resident in Paris. Professor Bouillaud is likewise reported to be suffering from a very severe attack of cholera.

---

DEATH OF PROFESSOR BARBOUR, OF ST. LOUIS.

It is with deep regret we have to record the decease—by the fatal epidemic which has rendered St. Louis desolate, and been fatal to so many of its exemplary citizens—of Professor Barbour, who ably filled the chair of Obstetrics in the University of Missouri, the medical department of which is located in St. Louis.

It is not more than two months since we saw him in this city full of health and enthusiasm in the profession of his choice; zealous in the pursuit of knowledge; and energetic in availing himself of every means of information to enable him to be more eminently useful in the responsible office of a teacher of the important branch assigned him in the medical school of which he was one of the ornaments.

Professor Barbour was a native of Virginia, and a son of Judge Barbour, of the Supreme Court of the United States, one of the distinguished worthies whose memory Virginia cherishes. He received his professional education partly at the University of Virginia, and graduated in the University of Pennsylvania. Many years ago he moved to the south-west, and occupied for some time, with marked credit, the chair of Chemistry, in Lagrange College, Tennessee. The chair of Obstetrics, in the University of Missouri, having been offered him, he moved to St. Louis some years ago, and has since resided there; honored as an able teacher; beloved as a man; extensively confided in as an honorable and philanthropic physician; and, when prematurely cut off, having still brighter prospects before him. He had the greatest confidence in a compound of morphia, Hoffmann's anodyne liquor, and other excitants, similar to many formulæ in use in cholera and choleroid affections; but his own case is another melancholy example of the utter inapplicability of any remedy, or combination of remedies, for special morbid conditions, which may, notwithstanding, have received a common appellation.

---

OBITUARY.—MR. CLIFT, F. R. S.

The last English steamer brought us the intelligence of the death of this gentleman. Mr. Clift has been long and deservedly well known, both in this country and in England, as the Conservator of the Hunterian Museum of the College of Surgeons, London; a situation which he has held for nearly half a century; as he was appointed to that office on the purchase (in 1799) of the collection by the British Government from the executors of John Hunter, whose pupil and assistant he was for many years. All will remember the part he took in the exposure of that literary incendiary, Sir Everard Home. The statement of the destruction of the Hunterian MSS. was obtained from him in his examination before a committee of the House of Commons, when this nefarious affair was first made public. Mr. Clift has been engaged for many years in preparing a *descriptive* catalogue of the Museum. Of those parts of the *general* catalogue which have been published, two, it is generally supposed, were drawn up by him,—the first and second, containing an account of the Pathological preparations in spirit and in a dry state; the rest being done by his late son, Mr. Home Clift, and his son-in-law, Prof. Owen.

---

CORRECTION.

The analysis of cod liver oil presented to our readers in the last No. of the Examiner, should have been credited to De Jongh, as published at Leyden, in 1843, in his "Disquisitio comparativa chemico-medics de tribus olei jecoris aselli speciebus;" a condensed account of which, by Dr. Pereira, will be found in the Record of the present number, under the head of *Chemistry*. The analysis is remarkable for its accuracy and completeness.

EDITORIAL AND OTHER CHANGES.

The New York Annalist has been discontinued, and its subscription list transferred to the New York Journal of Medicine. The late editor, Dr. N. S. Davis, has been appointed Professor of Physiology and Pathology in Rush Medical College, Chicago.

Two additional chairs have been created in the Medical College of Ohio, viz., Physiology and General Pathology, by Dr. L. M. Lawson, and Surgical Anatomy and Clinical Surgery, by Dr. I. T. Shotwell. Dr. Daniel Drake now occupies the chair of Special Pathology and Practice of Medicine, and Dr. G. W. Bayless, that of Descriptive Anatomy.

In the University of Louisville, Prof. Short has resigned the chair of Materia Medica, and Dr. Lewis Rogers appointed in his stead.

Dr. A. H. Baker, of Cincinnati, has been appointed Professor of Surgery in Indiana Central Medical College.

In Transylvania University, Prof. Annan has been transferred to the chair of Theory and Practice, and Prof. Wm. M. Boling, of Montgomery, Ala., appointed to that of Obstetrics, vacated by Prof. Annan.

The New Orleans Medical and Surgical Journal comes to us in a new dress, and with the gratifying announcement, that the sixth volume is commenced under more encouraging auspices than at any time since the work was commenced. We heartily congratulate our friends on their flattering prospects.

MEDICAL DEPARTMENT, U. S. ARMY.

The following gentlemen having passed a satisfactory examination at the recent meeting of the Board of Examiners in New York, have been appointed Assistant Surgeons in the Medical Department of the U. S. Army.

Wm. H. Ballard, of Louisiana; George K. Wood, of New York. Report in person at Jefferson Barracks.

Joseph P. Brown, of Michigan. Report in person at Fort Mackinac.

Alexander B. Hasson, of Maryland. Report in person at Fort Leavenworth.

Jonathan Letherman, of Pennsylvania. Report in person at Fort Monroe.

William A. Hammond, of Pennsylvania. Report in person at Carlisle Barracks, for duty with troops under orders to Santa Fe.

Francis Sorrell, of Georgia. Report in person at Fort Johnson, N. C.

Edward W. Johns, of Maryland. Report in person at Fort Columbus.

William W. Anderson, of South Carolina. Report in person at Fort McHenry.

---

### CHOLERA IN PHILADELPHIA.

The following table, from one of the daily prints, gives the entire number of interments of cholera and other diseases, from the time the disease first appeared, up to Saturday, the 28th ult.

| | *Cholera.* | *Other Diseases.* | *Total.* |
|---|---|---|---|
| Week ending June 2 - - - | 3 | 135 | 138 |
| " " " 9 - - - | 3 | 127 | 130 |
| " " " 16 - - - | 5 | 114 | 119 |
| " " " 23 - - - | 14 | 170 | 184 |
| " " " 30 - - - | 80 | 263 | 343 |
| " " July 7 - - - | 170 | 234 | 404 |
| " " " 14 - - - | 179 | 279 | 458 |
| " " " 21 - - - | 196 | 309 | 505 |
| " " " 28 - - - | 136 | 279 | 415 |
| Total, nine weeks, | 786 | 1910 | 2696 |

Of these 786 deaths by cholera, about 350 have occurred in the almshouse and in the cholera hospitals, leaving 436 that have occurred in private practice—a fraction more than one in a thousand of our population.

In New York the bills of mortality have reached an alarming height. The entire number of interments in that city during the week ending on July 21st, was 1409, and of these there were of cholera 714; which is 72 less than we have had in this city during the nine weeks that it has prevailed here. The following exhibits the number of interments in that city since the epidemic broke out:

| | *Cholera.* | *Other Diseases.* | *Total.* |
|---|---|---|---|
| Week ending May 26 - - - | 13 | 281 | 294 |
| " " June 2 - - - | 29 | 241 | 270 |
| " " " 9 - - - | 121 | 288 | 409 |
| " " " 16 - - - | 146 | 280 | 425 |
| " " " 23 - - - | 152 | 321 | 473 |
| " " " 30 - - - | 286 | 448 | 734 |
| " " July 7 - - - | 317 | 385 | 702 |
| " " " 14 - - - | 484 | 507 | 991 |
| " " " 21 - - - | 714 | 695 | 1409 |
| Total, nine weeks, | 2261 | 3446 | 5707 |

During the first nine weeks of the epidemic of 1832, the entire mortality in New York was 4673, and the deaths by cholera were 2999. The aggregate of this year exceeds that of 1832 considerably, and the deaths by cholera are rapidly coming up to the figures of that year, while the number of deaths from it during the week ending July 21st is within two of the highest weekly number of that year, which was 716. It must, however, be remembered that the population is immensely increased since that time, and the city is now crowded with immigrants, among whom a large proportion of the deaths occur.

---

CHOLERA IN THE BLOCKLEY ALMSHOUSE, PHILADELPHIA.

The number of inmates of all classes at the Philadelphia Almshouse, which at the outset of the Cholera invasion amounted to about 1700, was rapidly reduced by various causes, so as to range between 1400 and 1500 during the latter half of the period referred to. The average population of the establishment may be therefore estimated at about 1600 for the past eight weeks.

A comparison of this roughly approximative statement with the reports above presented will exhibit, at a glance, the serious extent to which the epidemic has recently prevailed within the Blockley precincts. Nor can it fail to force upon the notice of the reader the very grave mortality which has marked the progress of that fell disease among the pauper residents. Slight and gentle as the visitation to our city generally ought to be regarded, we must confess the mysterious enemy has found no lack of prey upon the debilitated frames of the unhappy pensioners of the public bounty. Still more alarmingly true is it that the disproportionate array of fatal cases has left a miserably narrow margin for exaggeration to the most terror-stricken dealer in the marvellous.

The Pennsylvania Hospital, Wills Hospital, Moyamensing Prison, Eastern Penitentiary, in a word, all our Asylums and houses of detention, have been in a great degree exempt, while the unlucky inhabitants of the County poor-house appear to have been stricken with a special doom.

What is the meaning of this dreadful and singularly marked exception to the general rule? Why is it that in all the abodes but one of the dissolute and destitute, the infirm, sick and disabled, the members of these classes continue to escape; while in that one, crowds of wretches are seized upon so fatally, that certainly not less than 80 out of every hundred thus attacked have sunk down and died at once in spite of early and well directed efforts on the part of medical attendants, whose skill and devotion no one properly informed will pretend to call in question? Simple justice to these medical officers requires a clear and full explanation of the facts as far as they are known. We have elsewhere spoken of the dangerous, and in all respects self-sacrificing services of these gentlemen of the Almshouse staff; and have been subjected to the painful duty of recording the ravages of death as well as of disease among them—almost the only appreciable result of their unflinching constancy and zeal in the discharge of a cheerfully accepted duty. Our object in this hurried note is to call for more satisfactory information than is yet within our reach, in relation to the supposed and alleged causes of a state of things in our opinion entirely too deplorable to be quietly observed without investigation in the proper quarter. In the face of a loss of life so seriously out of all proportion to the casualties in other similar assemblages infected with the same disorder, we cannot help suspecting that there has been "something rotten" in the jurisdiction of our Guardians of the Poor; and we consider a strict and fair account of the recent hygienic management of their unwieldly "institution," as well as of the miasmatic influence presumed to be at work around it, to be due not only to the members of the medical profession here, who have so much at stake in the inquiry, but to the whole community around us, if not to humanity at large.

We are willing to attribute much to the "want of stamina"—to use a favorite apologetic phrase—necessarily existing in the aged, diseased, and otherwise depressed and disabled patients whose already broken health inevitably swells the death-list of a poor-house ward; but we cannot forget that in these respects the denizens of the Blockley galleries and out wards, and even of the infirmary and lunatic asylum, are not a whit worse off than crowds of their vagrant compeers in other equally populous districts of this country, while they are decidedly superior as a class to the habitual frequenters of the Union workhouses and hospices abroad. Medical men, who are too familiar with the natural history of the pauper species to be content with such a meagre etiology, must look beyond the individual patient and his previous habits for a complete solution of the problem of his utter inability to resist the first onset of a new disease, albeit that messenger of death be cholera itself. We have reason to believe from some observation and inquiry of our own, and still more on the authority of information which has reached us from various sources entitled to respect, that the prevalence of the disease, as well as the appalling failure of the ordinary and established modes of treatment hitherto successful in a large number even of pauper cases, may be attributed in a considerable degree to errors of dietetic and other general management, which the physicians supposed to be entrusted with the direction of such matters, have not been permitted to correct. We have no doubt that a great change is needed, not only in the diet of the inmates of every department of the Blockley establishment, but in the regulation of their exercise and occupation, their personal cleanliness and other habits, and the proper purification, ventilation and other arrangements of their dormitories, dining halls and day rooms. We rejoice, therefore, that public attention has been attracted to the direction of its internal affairs; and whatever may be the merits of the family quarrel which has for some time past notoriously distracted and impeded the useful action of the directing Board, we are led to hope that the advocates of true progress have at length attained a permanent and efficient ascendency in the administrative power.

As an earnest of this better prospect, we take pleasure in stating that a committee of Guardians, together with the medical superintendent, have lately completed a tour of inspection of the principal asylums and similar houses of employment in the neighboring states. Since their return, these gentlemen have published a report of the result of their inquiries; and in the course of this they strongly urge the necessity of several important alterations, which we sincerely trust are destined ere long to prove but the prelude to an entire reform.

The report of the Almshouse commission has been received too late to admit of the notice which it certainly deserves; but much as we are pressed for time and space, we cannot close without particular allusion to the remarks of the reporters, upon the importance of allowing to the superintending resident-physician the "entire control and supervision" of the system of administration, for the results of which, from the nature of his office, he is held responsible. The absurd anomaly of a medical superintendent burthened with the responsibility of securing the welfare and safety of a large population, but unfurnished with authority to check mismanagement and enforce his own administration, one might suppose were not to be found in a rational community, much less in one pretending to the front rank of civilization and humanity. Here especially in Philadelphia, noted for her philanthropy and the elevated rank of her medical fraternity, such a strangely contradictory and mischievous position of a medical office of high trust and honor should hardly be allowed to stare us in the face; and yet we have it not only in the Blockley Almshouse, as a matter of indisputable notoriety and long established usage, but it may be seen in virtual operation in places of far wider note and much greater pretensions than a county poor-house. The common sense of every humane man must revolt at such an unjust and worse than ridiculous severance of means and ends. We cannot afford to dwell upon it now. The importance of the theme may be our warrant for a return to its consideration at another time. We may take occasion then to be a little more explicit in regard to more than one institution, in which we have reason to believe the recommendations of the responsible physician are habitually disregarded, to the manifest detriment, not only of his general usefulness, but of the health of the subjects supposed to be committed to his sole and untrammeled professional care.

